# Risk factor analysis of plastic bronchitis among 126 children with macrolide-resistant *Mycoplasma pneumoniae* pneumonia with mutations at the *A2063G* site after bronchoscopy examination: a nomogram prediction model

**DOI:** 10.3389/fped.2025.1521954

**Published:** 2025-02-27

**Authors:** Ran Ma, Ting Bai, Bo Yuan, Li Zhang, Shanshan Li, Lanhua Ma, Wei Zhang

**Affiliations:** ^1^ Pediatrics Department, The First Affiliated Hospital of Shihezi University, Shihezi, China; ^2^ Obstetrics Department, The First Affiliated Hospital of Shihezi University, Shihezi, China; ^3^ Pediatrics Department, Jining NO.1 People's Hospital, Jining, China

**Keywords:** children, plastic bronchitis, macrolide-resistant *Mycoplasma pneumoniae* pneumonia, *A2063G* site, nomogram model

## Abstract

**Objective:**

To determine the risk factors for plastic bronchitis (PB) in children diagnosed with macrolide-resistant *Mycoplasma pneumoniae* (MRMP) pneumonia associated with the *A2064G* variant.

**Methods:**

The clinical data of 126 children diagnosed with MRMP pneumonia (all with mutations at the *A2063G* site) who underwent bronchoscopy from May 2023 to April 2024 were retrospectively collected. Based on bronchoscopic findings, patients were classified into the PB and non-PB groups. The study compared the general and clinical features, laboratory indicators, imaging features, bronchoscopic manifestations, treatment, and prognosis between the two groups. A nomogram model, based on logistic regression, was developed to estimate the risks of developing PB in children with MRMP pneumonia caused by mutations at the *A2063G* site.

**Results:**

We included 68 boys and 58 girls in this study, with 32 (25.4%) belonging to the PB group. The nomogram model constructed in this study indicated that three risk factors—Atelectasis, *Mycoplasma pneumoniae* genome copies (throat swab) >10^5^, and D-dimer levels—could be used for the early identification of MRMP pneumonia-induced PB. The area under the receiver operating characteristic curve for the predictive model was 0.832 (95% confidence interval: 0.743–0.922). The Hosmer-Lemeshow goodness-of-fit test demonstrated good calibration of the nomogram (*P* = 0.227, R^2^ = 0.403). Decision curve analyses revealed that the model has clinical value. Regarding treatment, second-line drugs and the frequency of bronchoscopy were significantly higher in the PB group than in the non-PB group.

**Conclusions:**

Early risk factor identification and bronchoscopy can improve the outcomes of children with PB associated with MRMP pneumonia caused by mutations at the *A2063G* site.

## 1 Introduction

Plastic bronchitis (PB), which is diagnosed via bronchoscopy, is a rare lung disease in children. It is characterized by the formation of rubbery, twig-like tubes that partially or completely obstruct the airway, leading to atelectasis, progressive dyspnea, and potentially respiratory failure or death (1–3). PB can occur in children of all ages (4). The number of diagnosed PB cases is gradually increasing, potentially due to the extensive development and use of bronchoscopy in recent years. PB has been reported in cases involving respiratory infection, the Fontan operation, cystic fibrosis, and allergic diseases (2, 5, 6). *Mycoplasma pneumoniae* (MP) is one of the most important pathogens causing lower respiratory tract infections in school-age children (7). In recent years, there have been increasing reports of children with macrolide-resistant *Mycoplasma pneumoniae* (MRMP) pneumonia complicated with PB (8), and some children have developed sequelae as a result of it (9, 10). The pathogenesis of PB may be related to the following factors: MRMP infection, excessive body temperature and fluid loss leading to sticky secretions, and an exaggerated immune response, causing severe airway injury and ciliary clearance dysfunction (11–13). Currently, predicting how PB occurs in children with MRMP pneumonia and avoiding adverse outcomes are the key issues for clinicians. In this study, we identified the risk factors for PB induced by MRMP pneumonia and developed a nomogram to enable the early identification of PB based on clinical features. This nomogram facilitates timely bronchoscopic cast removal, thereby accelerating patient recovery and reducing the proportion of MRMP pneumonia cases with a poor prognosis.

## 2 Materials and methods

### 2.1 Study subjects

**Figure 1:**
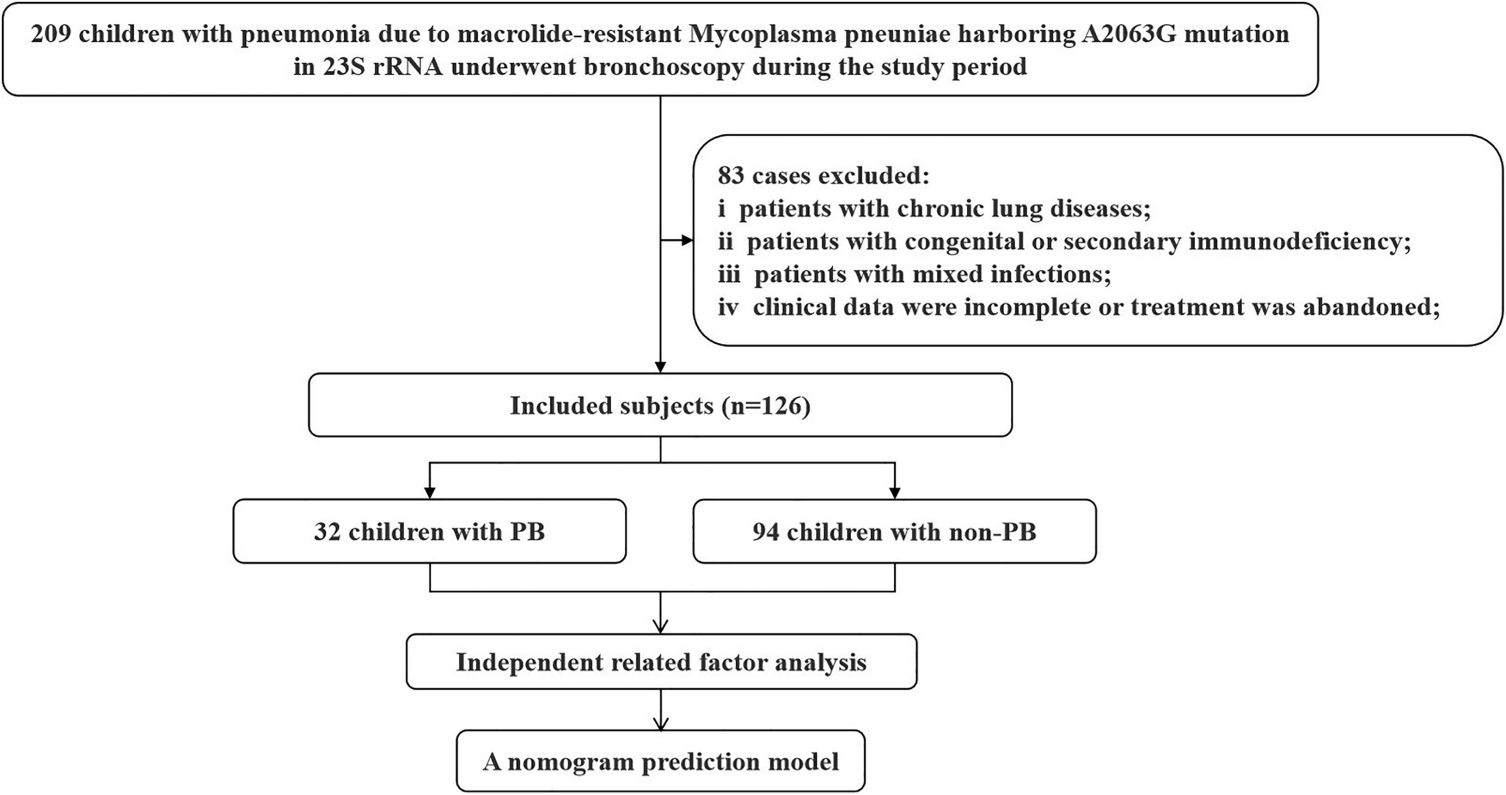
Flow diagram of the participant inclusion process.

There were 126 children aged 1 month to 14 years who were admitted to the hospital and diagnosed with MRMP pneumonia after bronchoscopy at the First Affiliated Hospital of Shihezi University from May 1, 2023, to April 30, 2024 (Figure 1). Prior to admission, all the children tested positive for serum MP antibodies. Written informed consent was obtained from the parents or guardians of all the children.

The diagnostic criteria for MRMP pneumonia were based on the “Guidelines for Diagnosis and Treatment of *Mycoplasma pneumoniae* Pneumonia in Children (2023 Edition)” (14) and the “Expert Consensus on the Diagnosis and Treatment of Macrolide-Resistant *Mycoplasma pneumoniae* Pneumonia in Children” (15). All participants met the following inclusion criteria: (i) Presentation of signs and symptoms of pneumonia, such as fever, cough, or the presence of dry and wet rales; (ii) Lung imaging showing evidence of inflammatory lesions; (iii) Undergoing bronchoscopy; (iv) Testing positive for MP-DNA and the presence of a resistance gene mutation at the *A2063G* site. We excluded the following categories of patients: patients with chronic lung diseases; those with congenital or secondary immunodeficiency; those with mixed infections; and those with incomplete clinical data or who abandoned treatment.

### 2.2 Grouping

**Figure 2:**
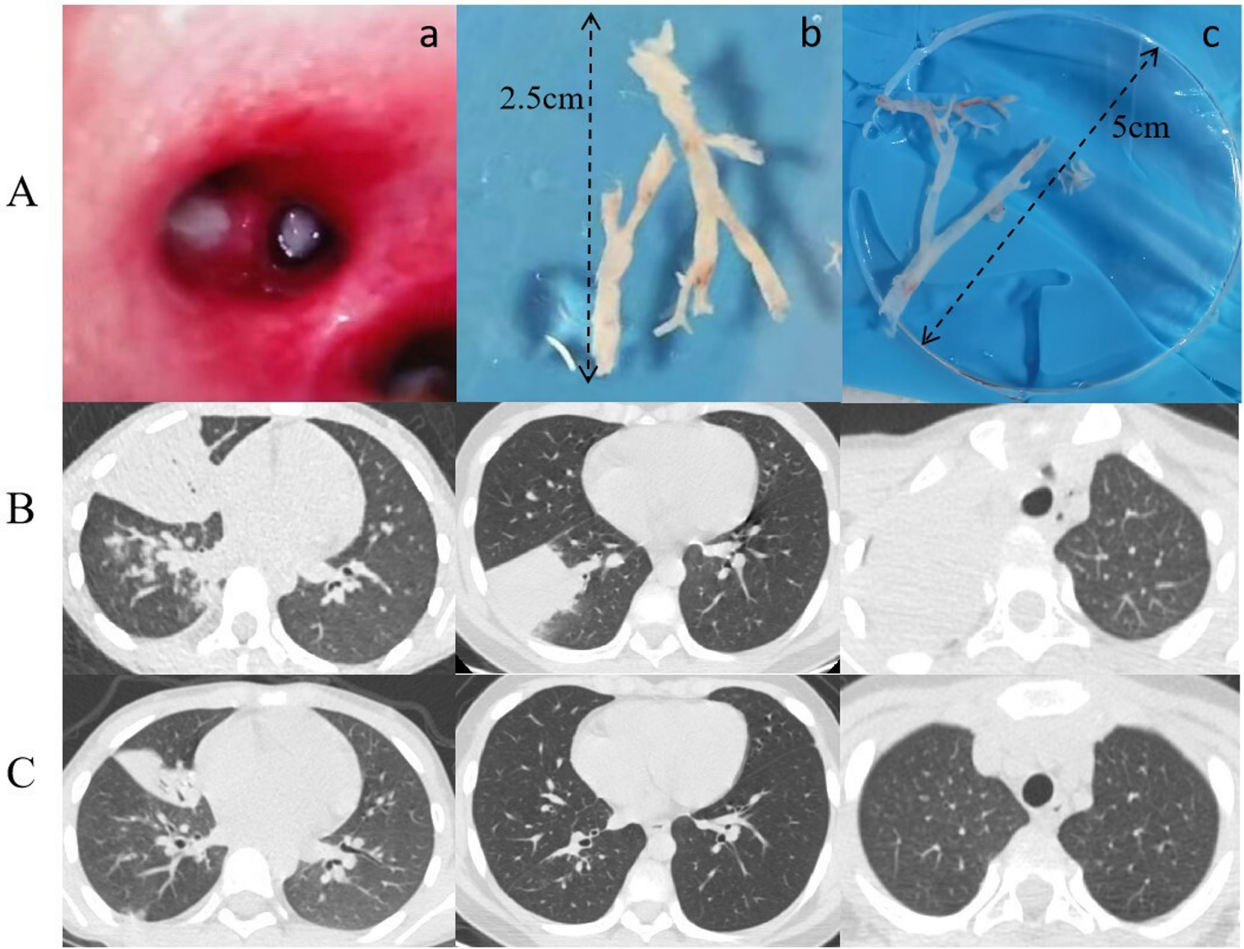
**(A)** **a**: Imaging of bronchial casts prior to bronchoscopy procedure; **(b, c)** bronchial casts removed under bronchoscopy. **(B)** Lung CT Scan: Pre-bronchoscopy assessment. **(C)** Lung CT Scan: Post-bronchoscopy status.

Based on whether the bronchoscopy findings revealed bronchial casts (Figure 2A), 32 cases were classified into the PB group and 94 into the non-PB group.

### 2.3 Clinical data collection

The following clinical data were collected: general and clinical features, laboratory indicators, imaging features, bronchoscopic manifestations, and treatment and prognosis. When the clinical manifestations disappeared or the patient recovered, the treatment was deemed effective.

### 2.4 Etiological testing

On the day of admission, pharyngeal swabs were collected for the detection of 107 respiratory pathogens by targeted Next-Generation Sequencing (tNGS; see Supplementary Table 1) at Jinyu Medical Laboratory in Guangzhou, China. The tNGS panel included the detection of MP mutation loci. Within three days of admission, a bronchoscopy was performed to obtain bronchoalveolar lavage fluid (BALF) for the determination of MP genome copy numbers.

### 2.5 Treatment

Clinicians select antibiotics based on the “Guidelines for Diagnosis and Treatment of *Mycoplasma pneumoniae* Pneumonia in Children (2023 Edition)” and the “Expert Consensus on the Diagnosis and Treatment of Macrolide-Resistant *Mycoplasma pneumoniae* Pneumonia in Children” (14, 15). According to these guidelines, first-line antimicrobial agents for the treatment of MP pneumonia include erythromycin, azithromycin, and clindamycin. If fever persists or chest imaging demonstrates continued progression of inflammatory lesions after 48–72 h of macrolide therapy, clinicians are advised to consider switching to second-line antimicrobial agents. The recommended second-line drugs include doxycycline and levofloxacin, which have demonstrated effectiveness against macrolide-resistant strains of MP. Intravenous immunoglobulin (IVIG) therapy for MRMP pneumonia is not routinely recommended; however, it may be considered in cases of severe extrapulmonary complications like central nervous system damage, skin/mucous membrane lesions, hematological issues, or others.

During hospitalization, bronchoscopy was conducted in accordance with the “Guideline of Pediatric Flexible Bronchoscopy in China (2018 Edition)” (16). All enrolled children underwent bronchoscopy with BALF collection within three days of admission. Informed consent was obtained for all treatment plans. All data originated solely from past cases, with no additional interventions. Consequently, no clinical trials were implicated in this study.

### 2.6 Statistical analysis

SPSS software (V27.0, IBM, New York, USA) and R software (V.4.4.2, R Foundation for Statistical Computing, Vienna, Austria) were used for all statistical analyses. Continuous variables are presented as mean [95% confidence interval (CI)] and compared between two groups using *t*-tests. Categorical data are expressed as frequencies with percentages and compared between groups using the *χ*2 test or Fisher's exact test. *P* < 0.05 was considered statistically significant. Exposure variables that were significantly associated with the outcome in the univariate logistic regression analysis were included in a stepwise multivariate logistic regression analysis. Based on the results of the previous multivariate analysis, a nomogram was constructed. The discrimination and calibration of the nomogram were evaluated using the area under the receiver operating characteristic curve (AUC), the Hosmer-Lemeshow goodness-of-fit test (HL test), and the calibration plot. The decision curve analysis (DCA) was performed to assess the clinical usefulness of the predictive models.

## 3 Results

### 3.1 General situation and clinical features

There were 126 children in total, with 32 (25.4%, comprising 17 boys and 15 girls) in the PB group. Among the 32 children in the PB group, there was 1 toddler, 6 children of preschool age, and 25 children of school age. Additionally, 7 children were admitted in summer, 5 in autumn, and 20 in winter. There were no significant differences in peak temperature, length of hospital stay, clinical manifestations, pulmonary examination findings, and respiratory complications between the PB and non-PB groups (Table 1).

**Table 1:** Sociodemographic characteristics of our study population.

Variables	All (*N* = 126)	PB (*N* = 32)	Non-PB (*N* = 94)	*P*-value
Sex, *n* (%)				0.912
Boys	68 (54.0)	17 (53.1)	51 (54.3)	
Girls	58 (46.0)	15 (46.9)	43 (45.7)	
Age, year	12.45 (3.65*–*21.24)	8.22 (7.47*–*8.98)	13.88 (2.06*–*25.71)	0.900
Hospitalization (d)	9.36 (8.75*–*9.97)	9.91 (8.84*–*10.97)	9.17 (8.84*–*9.91)	0.087
Peak temperature (℃)	39.59 (39.46*–*39.73)	39.73 (39.50*–*39.95)	39.55 (39.39*–*39.71)	0.252
Cough, *n* (%)	126 (100.0)	32 (100.0)	94 (100.0)	
Rale, *n* (%)	87 (69.0)	24 (75.0)	63 (67.0)	0.399
Hypopnea, *n* (%)	11 (8.7)	2 (6.3)	9 (9.6)	0.728
Dyspnea, *n* (%)	11 (8.7)	2 (6.3)	9 (9.6)	0.728
Hypoxemia, *n* (%)	20 (15.9)	6 (18.8)	14 (14.9)	0.606
External respiratory complications, *n* (%)				
Otalgia	2 (1.6)	0 (0.0)	2 (2.1)	0.630
Rash	4 (3.2)	1 (3.1)	3 (3.2)	1.000
Thoracodynia	2 (1.6)	1 (3.1)	1 (1.1)	0.445
Gastrointestinal symptoms	4 (3.2)	0 (0.0)	4 (4.3)	0.571
Abnormal liver function	32 (25.4)	1 (37.5)	20 (21.3)	0.069
Heart failure	1 (0.8)	0 (0.0)	1 (1.1)	1.000

Abbreviations: PB, plastic bronchitis. Significant *P*-value are bold, *P* < 0.05.

Continuous variables are presented as the mean [95% confidence interval (CI)].

### 3.2 Laboratory indicators and etiological results

The percentage of neutrophils, as well as the levels of lactate dehydrogenase, creatine kinase isoenzyme and D-dimer in routine blood samples collected from children in the PB group were significantly higher than those in samples collected from children in the non-PB group (*P* = 0.016, P<0.001, *P* = 0.049, P<0.001, respectively). The number of MP genome copies (detected via throat swab) was significantly higher in the PB group than in the non-PB group (*P* = 0.005). The incidence of atelectasis was also significantly higher in the PB group than in the non-PB group (*P* < 0.001). However, there were no statistically significant differences in the time to fever resolution and the incidence of adverse reactions after bronchoalveolar lavage between the two groups (Table 2).

**Table 2:** Comparison of lab test and radiological features.

Variables	All (*N* = 126)	PB (*N* = 32)	Non-PB (*N* = 94)	*P*-value
Lab test				
WBC (×10^9^/L)	8.77 (7.23–10.30)	7.19 (6.30–8.08)	9.30 (7.28–11.33)	0.208
NEUT %	66.21 (64.11–68.30)	69.78 (66.50–73.07)	64.99 (62.42–67.55)	**0** **.** **016**
Hb (×10^9^/L)	121.10 (122.83*–*119.37)	119.56 (116.32*–*122.80)	121.63 (119.56*–*123.69)	0.306
PLT (×10^9^/L)	266.98 (253.67*–*280.30)	250.38 (224.03–276.72)	272.64 (257.10–288.18)	0.097
CRP (mg/L)	40.65 (30.95*–*50.36)	34.15 (16.38–51.92)	42.87 (31.21–54.53)	0.606
Procalcitonin (ng/ml)	1.12 (0.30*–*1.93)	0.35 (0.20–0.51)	1.37 (0.29–2.46)	0.128
IL-6 (pg/ml)	41.30 (30.07*–*52.52)	47.67 (28.11–67.23)	39.13 (25.46–52.79)	0.341
Lactate dehydrogenase (U/L)	373.57 (347.50*–*399.65)	411.50 (383.27–499.73)	350.45 (322.54–378.36)	**<0**.**001**
Creatine kinase isoenzyme (U/L)	22.88 (19.34*–*26.42)	29.36 (16.00–42.73)	20.68 (19.03–22.32)	**0**.**049**
D-dimer (mg/L)	3.11 (2.10*–*4.12)	5.86 (2.07–9.65)	2.18 (1.77–2.58)	**<0**.**001**
*Mycoplasma pneumoniae* genome copies (throat swab), *n* (%)				**0.005**
>10^5	100 (79.4)	31 (96.9)	69 (73.4)	
≤10^5	26 (20.6)	1 (3.1)	25 (26.6)	
Pulmonary radiological features, *n* (%)				
≥2 lung lobes	97 (77.0)	25 (78.1)	72 (76.6)	0.859
Consolidation	83 (65.9)	24 (75.0)	59 (62.8)	0.207
Atelectasis	32 (25.4)	18 (56.5)	14 (14.9)	**<0**.**001**
Pleural adhesions	6 (4.8)	2 (6.3)	4 (4.3)	0.643
Pleural thickening	9 (7.1)	2 (6.3)	7 (7.4)	1.000
Pleural effusion	49 (38.9)	16 (50.0)	33 (35.1)	0.136

Abbreviations: PB, plastic bronchitis. Significant *P*-value are bold, *P* < 0.05.

Continuous variables are presented as the mean [95% confidence interval (CI)].

### 3.3 Treatment and bronchoscopy

When comparing the treatment status of the participants in the two groups (Table 3), we found that the utilization rate of second-line antibiotics (doxycycline and levofloxacin) in the PB group was significantly higher than that in the non-PB group (*P* = 0.008). However, there was no significant difference in the utilization rate of IVIG and glucocorticoids (GCs) between the two groups. The number of alveolar lavages in the PB group was significantly higher than that in the non-PB group (*P* = 0.015). During the follow-up period, one child in the PB group with residual atelectasis underwent bronchoscopy twice during the course of their disease (Figures 2B,C).

**Table 3:** Comparison of treatments.

Variables	All (*N* = 126)	PB (*N* = 32)	Non-PB (*N* = 94)	*P*-value
Antibiotic, *n* (%)				**0** **.** **040**
First-line drug	75 (59.5)	14 (43.8)	61 (64.9)	
Second- line drug	51 (40.5)	18 (56.2)	33 (35.1)	
IVIG, *n* (%)	6 (4.8)	2 (6.3)	4 (4.3)	0.643
Glucocorticoids, *n* (%)	5 (46.8)	19 (59.4)	40 (42.6)	0.100
No. of alveolar lavages, *n* (%)				**0.015**
1 time	123 (97.6)	29 (90.6)	94 (100.0)	
2 times	3 (2.4)	3 (9.4)	0 (0.0)	

Abbreviations: PB, plastic bronchitis; IVIG, intravenous immunoglobulin. Bold values, significant at *P* < 0.05.

### 3.4 A nomogram for predicting the occurrence of PB in children with MRMP pneumonia with mutations at the *A2063G* site

After excluding confounding factors, the multivariate stepwise logistic regression analysis showed that the D-dimer level was an independent risk factor for PB in children with MRMP pneumonia (Table 4). The receiver operating characteristic (ROC) curve analysis indicated that the D-dimer level of 3.68 mg/L, with an AUC of 0.715, represents the optimal threshold for diagnosing children with MRMP pneumonia.

**Table 4:** Multifactorial stepwise logistic regression analysis.

Variables	β	S.E.	Wald	*p*-value	OR	95% CI
Atelectasis	2.097	0.523	16.095	**<0**.**001**	8.142	2.923–22.680
*M. pneumoniae* genome copies (throat swab) >10^5^	2.387	1.085	4.835	**0**.**028**	10.879	1.296–91.308
D-dimer	0.246	0.092	7.153	**0**.**007**	1.279	1.068–1.531

The D-dimer level was identified as an independent risk factor for PB. PB, plastic bronchitis. Significant *P*-value are bold, *P* < 0.05.

**Figure 3:**
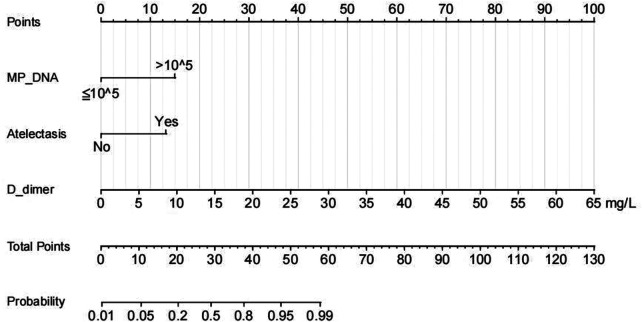
Nomogram of regression equations for calculating the risk score and predicting the risk of PB in children with MRMP pneumonia with mutations at the *A2063G* site.

**Figure 4:**
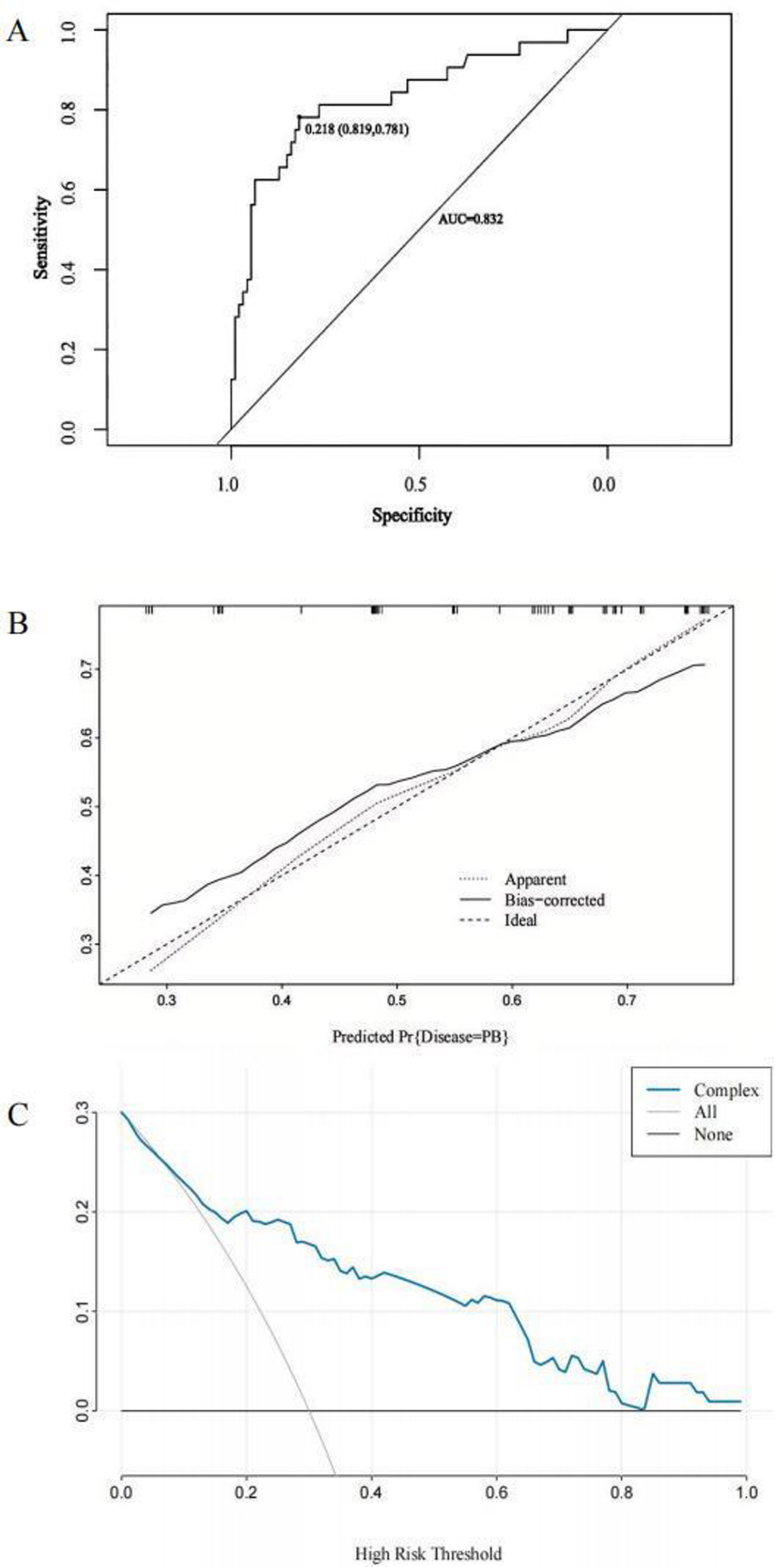
Receiver operating characteristic (ROC) curves, calibration curves, and decision curve analysis (DCA) to predict PB in children with MRMP pneumonia at the *A2063G* site. **(A)** The ROC curve analysis of the main cohort. **(B)** Calibration curves for the predicted nomogram. The horizontal axis indicates the risk of PB occurrence predicted by the nomogram, while the vertical axis represents the actual observed risk of PB occurrence based on the variables included in the nomogram [atelectasis, MP genome copies [throat swab) >10^5^, and the D-dimer level]. **(C)** DCA for the nomogram model. The DCA shows the clinical usefulness of the nomogram, and the *y*-axis indicates the net benefit. The straight line represents the assumption that all patients with MRMP pneumonia having mutations at the *A2063G* site will develop PB, and the horizontal line represents the assumption that no patient will develop PB.

A nomogram for predicting the risk of PB was constructed based on the three significant risk factors identified through the logistic regression analysis (Figure 3). Each independent influencing factor was assigned a weighted score, with the maximum possible score being 100 points. The predicted probability of PB incidence ranges from 0.01 to 0.99. Higher scores, which are derived from the cumulative distribution points of each high-risk factor, indicate a greater likelihood of PB occurrence. The HL test was adopted for the model test, and the result was *P* = 0.227, R^2^ = 0.403, indicating that the information in the current data had been fully extracted. The AUC value showed that the predictive power of the predictive model in the main cohort was 0.832 (95% CI: 0.743–0.922) as shown in Figure 4A. The calibration chart showed that the nomogram had sufficient fit for predicting PB incidence in patients with MRMP pneumonia (Figure 4B). The DCA shows the clinical usefulness of the nomogram, as shown in Figure 4C. Our model gained significant clinical benefit.

## 4 Discussion

The extensive development of bronchoscopy technology has led to a gradual increase in the number of reports related to PB (2, 6, 17, 18). PB, a complication of pulmonary disease in children, is typically diagnosed through bronchoscopy, which reveals that the bronchial lumen is obstructed by a large, branched mucous plug. PB has been reported in patients who have undergone Fontan surgery, as well as in those with infections, cystic fibrosis, and allergic conditions (2, 5, 6). MP is a common cause of respiratory infections in children with PB. Occasionally, PB caused by the Influenza virus, Adenovirus, Respiratory Syncytial Virus, and *Haemophilus influenzae* has also been reported (1, 4, 19–21). There are two types of endobronchial casts in PB, type I and type II, with the former being typically associated with inflammatory diseases. The inflammatory cells in the tubular type of endobronchial casts are predominantly neutrophils (22). Pathological smears of BALF from all PB patients in our study indicated that neutrophils were the primary inflammatory cells, classifying the PB as type I.

MP is an important cause of PB (4), although the pathogenesis of PB caused by MP infection remains unclear. Zhan et al. suggest that children with MRMP pneumonia exhibit a more pronounced inflammatory response, which increases their likelihood of developing PB (12). Previous studies have reported that the primary mutation sites in the domain V of the 23S rRNA gene of MP are *A2063G*, *A2064G*, *A2067G*, and *C2617G* (23, 24). In our study, we detected MP resistance genes in children with PB, and the mutation site identified in all the macrolide resistance genes at this time was *A2063G*. Although no correlation was observed between the occurrence of MRMP pneumonia-induced PB and sex in our study, a correlation was found with age. The average age of the participants in the PB group was 8.22 years, and the incidence of PB was higher among older children, which may be attributed to the stronger immune response in this age group following an infection.

Bronchoscopy has established that the presence of casts in the airways serves as the gold standard for PB diagnosis and is also a critical therapeutic method for removing plastic secretions (2, 9, 25). However, bronchoscopy is an invasive procedure that can be particularly challenging for children with early-stage PB, and diagnostic delays may exacerbate the disease. Consequently, it is essential to identify the risk factors associated with PB in children with MRMP pneumonia while avoiding excessive drug use. Therefore, we compared the clinical manifestations, indicators, treatments, and outcomes of the two groups of children: those with PB and those without PB, and explored the risk factors for PB development. Our findings revealed that high fever was an important feature of MRMP pneumonia in children with PB. The average peak temperature among PB-affected children was 39.73℃, which is consistent with the findings of Zhao et al. (11). This may be attributed to the inflammatory response triggered by MP infection leading to an elevation in body temperature, and the viscous secretions resulting from dehydration facilitating PB formation. However, no significant differences were observed in dyspnea (including shortness of breath and hypoxemia), pulmonary signs (such as pulmonary rales and decreased breath sound), and extrapulmonary manifestations (such as rash and chest pain) between the two groups, perhaps due to the small sample size. Children with MRMP pneumonia associated with PB often exhibit atypical laboratory findings (11, 26). Further comparisons revealed that the D-dimer and Lactate dehydrogenase levels in the PB group were significantly higher than those in the non-PB group, suggesting that these two may serve as risk factors for PB development in children with MRMP pneumonia. Additionally, we observed that the number of MP genome copies in throat swabs of 96.9% of PB-affected children exceeded 10^5^, which was significantly higher than in the non-PB group. Pulmonary imaging aids in identifying the occurrence of PB, and the incidence of atelectasis and pleural effusion in children with PB is significantly increased (11, 26). In our study, atelectasis was observed in 56.5% of participants in the PB group. The multivariate logistic regression analysis showed that D-dimer levels, the number of MP genome copies (pharyngeal swab), and atelectasis were independent predictors of PB development in children with MRMP pneumonia. The ROC analysis for D-dimer levels suggested that a threshold of 3.68 mg/L may serve as a critical indicator of PB in children with MRMP pneumonia.

According to guidelines, doxycycline and levofloxacin are considered second-line treatment for children with MRMP pneumonia (14). In our study, the utilization rate of second-line drugs in children with MRMP pneumonia combined with PB was 56.2%, which was significantly higher than the 35.1% observed in the non-PB group. Additionally, three children (9.4%) underwent bronchoscopy twice, which is consistent with the findings reported by Huang et al. (4). Patients with PB may experience adverse outcomes such as atelectasis, bronchiolitis obliterans, and even death (10). However, 32 children with PB included in our study underwent bronchoscopy and active antibiotic treatment within three days of admission, and no deaths were reported. This indicates that early active PB treatment is helpful in reducing adverse outcomes.

Nevertheless, our study had some limitations. We only detected MP 23S rRNA mutation sites *A2063G*, *A2064G*, *A2067G,* and *C2617G*, potentially overlooking other drug resistance mutation sites or new drug resistance genes. Meanwhile, the sample size of our study was limited, and we did not perform either internal or external validation. Therefore, it will be necessary to increase the sample size to explore the risk factors for PB in children with MRMP further. Moreover, the retrospective nature of this study may have introduced some selection bias, implying that our findings require further validation by large-sample prospective studies.

## 5 Conclusion

In summary, PB was observed in 25.4% of pediatric patients with MRMP pneumonia. Although patients with early PB may exhibit no specific symptoms, laboratory results, or imaging findings, early bronchoscopy for the assessment and removal of plastic secretions is effective for relieving airway obstruction and mitigating the severity of the condition. In this study, we developed a nomogram with three factors, including atelectasis, the number of MP genome copies (throat swab) >10^5^, and the D-dimer level, to predict the risk of PB due to MRMP pneumonia in children with the *A2063G* mutation. On evaluating the accuracy and clinical application value of this model, we found that it is helpful for the early screening of high-risk groups in clinical practice and the implementation of intervention measures to reduce the risk of a poor prognosis.

## Data Availability

The raw data supporting the conclusions of this article will be made available by the authors, without undue reservation.
