# Supplementary material for: Risk factor analysis of plastic bronchitis among 126 children with macrolide-resistant Mycoplasma pneumoniae pneumonia with mutations at the A2063G site after bronchoscopy examination: a nomogram prediction model
Source: Front Pediatr. 2025 Feb 27;13:1521954. doi: 10.3389/fped.2025.1521954 (PMC11903698; doi:10.3389/fped.2025.1521954)
Supplement: Supplementary file 1 [file Table1.docx]

Supplementary table 1:

| 107 respiratory pathogens targeted metagenomic sequencing | | |
| --- | --- | --- |
| Virus | Bacteria | Atypical pathogens |
| Human adenoviruses | Cryptobacillus haemolyticus | Mycoplasma pneumoniae |
| Human adenovirus group A | Corynebacterium diphtheriae | Chlamydia pneumoniae |
| Human adenovirus group B | Staphylococcus aureus | Chlamydia psittaci |
| Human adenovirus group C | Streptococcus agalactis | Chlamydia trachomatis |
| Human adenovirus group D | Streptococcus galactosus | Mycoplasma hominis |
| Human adenovirus group E | Streptococcus intermedius | Mycoplasma genitalium |
| Human adenovirus type 1 | Streptococcus pneumoniae | Ureaplasma Urealyticum |
| Human adenovirus type 2 | Streptococcus pyogenes | Ureaplasma parvos |
| Human adenovirus 3 | Listeria monocytogenes |  |
| human adenovirus 4 | Streptococcus pharyngitis |  |
| Human adenovirus 5 | Corynebacterium ulcers |  |
| Human adenovirus 7 | Acinetobacter baumannii |  |
| Human adenovirus 8 | Bordetella pertussis |  |
| Human adenovirus 12 | Clostridium |  |
| Human adenovirus 18 | Haemophilus influenzae type A |  |
| Human adenovirus 21 | Haemophilus influenzae type B |  |
| Human adenovirus 24 | Klebsiella pneumoniae |  |
| Human adenovirus 27 | Legionella pneumophila |  |
| Human adenovirus 28 | Moraxella catarrhalis |  |
| Human adenovirus 30 | Neisseria gonorrhoeae |  |
| Human adenovirus 31 | Neisseria meningitidis |  |
| Human adenovirus 34 | Pseudomonas aeruginosa |  |
| Human adenovirus 38 | Serratia marcescens |  |
| Human adenovirus 55 | Stenotrophomonas maltophilia |  |
| Human herpesvirus 1 | Yersinia enterocolitica |  |
| Human herpesvirus 2 |  |  |
| Human herpesvirus 3 |  |  |
| Human herpesvirus 4 |  |  |
| Human herpesvirus 5 |  |  |
| Human herpesvirus 6 |  |  |
| Human herpesvirus 7 |  |  |
| Human Boca virus type 1 |  |  |
| Human parvovirus B19 |  |  |
| Enterovirus |  |  |
| Enterovirus A |  |  |
| Enterovirus B |  |  |
| Enterovirus C |  |  |
| Enterovirus D |  |  |
| Enterovirus A71 |  |  |
| Enterovirus D68 |  |  |
| Coxsackievirus A5 |  |  |
| Coxsackievirus A6 |  |  |
| Coxsackie virus A10 |  |  |
| Coxsackie virus A16 |  |  |
| Eccovirus 18 |  |  |
| Human coronavirus 229E |  |  |
| Human coronavirus HKU1 |  |  |
| Human coronavirus NL63 |  |  |
| Human coronavirus OC43 |  |  |
| Human metapneumovirus |  |  |
| Human respiratory syncytial virus A |  |  |
| Human respiratory syncytial virus B |  |  |
| Two genotypes of novel coronavirus (Alpha, Omicron) | |  |
| Human parainfluenza virus 1 |  |  |
| Human parainfluenza virus 2 |  |  |
| Human parainfluenza virus 3 |  |  |
| Human parainfluenza virus 4 |  |  |
| Influenza A virus |  |  |
| Influenza A virus H1N1 |  |  |
| Influenza A virus H3N2 |  |  |
| Influenza A virus H5N1 |  |  |
| Influenza A virus H7N9 |  |  |
| Influenza A virus H1N12009 |  |  |
| Influenza B virus |  |  |
| Influenza C virus |  |  |
| Measles virus |  |  |
| Mumps virus |  |  |
| Rhinovirus |  |  |
| Rhinovirus A |  |  |
| Rhinovirus B |  |  |
| Rhinovirus C |  |  |
| Rubella virus |  |  |
| Mycoplasma pneumoniae macrolide resistance gene / locus: 23SrRNA: A2063G, 23SrRNA: A2064G, 23SrRNA: A2067G, 23SrRNA:C2617G. | | |
